# Development and clinical assessment of new objective adherence markers for four microbicide delivery systems used in HIV prevention studies

**DOI:** 10.1186/s40169-018-0213-6

**Published:** 2018-11-07

**Authors:** Terry A. Jacot, Meredith R. Clark, Oluwatosin E. Adedipe, Susan Godbout, Abby G. Peele, Susan Ju, Jill L. Schwartz, Andrea R. Thurman, Gustavo F. Doncel

**Affiliations:** 1 Eastern Virginia Medical School, CONRAD, 601 Colley Avenue, Norfolk, VA 23507 USA; 2 CONRAD, 1911 North Fort Meyer Drive, Arlington, VA 22209 USA

**Keywords:** Adherence, HIV, PrEP, placebo, Spectroscopy, Microbicides

## Abstract

**Background:**

Adherence is critical for successful topical, vaginally delivered anti-retroviral (ARV)-based HIV pre-exposure prophylaxis (PrEP). Quantitating systemic or tissue ARV levels through LC–MS/MS is currently viewed as the most reliable measure of adherence. However, for placebo-controlled trials, this is a high cost analysis that measures adherence only in the drug treatment group. A desirable marker of adherence is one that is measured in both placebo and drug treatment groups using a simple on-site clinical laboratory test, which allows necessary interventions for supporting participant adherence. Our objective was to develop adherence markers for four vaginal placebo products currently used as microbicide delivery systems: gel, film, insert, and intravaginal ring. Excipient and spectroscopy-based approaches were used for preclinical development of the placebo markers and subsequently validated by the CONRAD 135 study. The study collected vaginal swabs collected each day for 1 week post vaginal application of gel, film, or insert in the clinic with or without sex. Intravaginal rings were collected after 1 day, 7, and 30 days of use.

**Results:**

Placebo gel, film, and insert in vaginal swabs were successfully detected by specific excipient colorimetric or probe-based assays for hydroxyethylcellulose, glycerin, and sorbitol respectively, as well as spectroscopy-based prediction models. The range of detection for gel, film, and insert in swabs collected up to 16 h post vaginal application was 70-100% of the total swabs per time point, with some markers showing potential for longer duration. Decreasing residual glycerin levels and increasing bioanalyte penetration of vaginally used intravaginal rings showed significant changes between 1 and 30 days of use.

**Conclusions:**

We demonstrated clinical proof-of-concept that adherence markers for placebo product can be measured using simple, lower cost approaches. Measuring adherence in both placebo and drug arms of a HIV PrEP study would better inform future trial designs.

**Electronic supplementary material:**

The online version of this article (10.1186/s40169-018-0213-6) contains supplementary material, which is available to authorized users.

## Background

It is well established that ARV-based PrEP for HIV prevention is successful when adherence is high [1]. However, previous HIV PrEP trials also make it clear that high adherence is difficult to achieve [2–7]. Since self-reports may overestimate adherence, due to social desirability or recall bias, much effort has been expended on finding ways to measure it objectively so as to improve participant compliance and analysis of the clinical trial results. The adherence measure that currently stands as the gold standard is systemic active drug levels quantitated by liquid chromatography tandem mass spectrometry (LC–MS/MS) [8]. Because of its high specificity and sensitivity, it provides highly accurate data, showing the discrepancy between self-report and actual drug adherence [9]. However, LC–MS/MS is costly and requires specialized technical personnel, equipment, and sample processing. In addition, drawing blood is an invasive procedure which some individuals are unwilling to undergo. Other less invasive sampling for LC–MS/MS has been investigated using hair, saliva, dried blood spots, and most recently urine [10–13]. Similar to other objective measures, it is typically performed at the end of a study when adherence interventions are often too late. A highly desirable marker of adherence would be a relatively simple, affordable, point of care test that could be readily processed by clinic personnel; immediate results would allow for implementation of interventions to increase PrEP adherence, as needed [14]. Lastly, LC–MS/MS analysis can only be done on samples from the treatment group of a placebo-controlled study. If adherence has a crucial effect on effectiveness, to measure it in both control and treatment groups would provide more accurate assessments. Subgroup analysis of effectiveness based on adherence in the active arm may be biased by differences in risk and HIV exposure. Therefore, our objective was to determine whether it was feasible to develop drug independent (placebo) markers using simple, low cost approaches for vaginally administered products.

To complete this objective we focused on topical, placebo vaginal dosage forms currently in development as microbicide delivery systems. The four delivery systems used for investigating markers of product adherence were the “universal” HEC (hydroxyethylcellulose) placebo gel [6, 7, 15, 16] and the placebo forms of the tenofovir (TFV) vaginal film [17], vaginal insert [18], and the dual reservoir TFV-levonorgestrel intravaginal ring (IVR) [19, 20]. Two main approaches for placebo marker development were investigated: 1) Excipient based detection methodologies and 2) spectroscopy methodologies, specifically Attenuated Total Reflectance Fourier Transform Infrared Spectroscopy (ATR-FTIR) coupled with chemometric modeling. Initial work for developing FTIR-based prediction models to determine the presence of placebo products in this report has been completed [21]. Once methodologies were developed to detect placebo product through excipient-based assays and FTIR spectroscopy, a clinical study was performed to determine feasibility and how long after vaginal use the product could be detected using the developed approaches. We previously developed biomarkers of semen exposure in the vagina and described the decay curves of these biomarkers [22, 23]. Since vaginal products will be used in the presence or absence of semen, the ability to detect the adherence markers in the presence of semen was also tested.

## Methods

### Vaginal dosage forms

Placebo vaginal insert was manufactured by CoreRx Inc. (Clearwater, FL) under cGMP. Placebo vaginal gel (HEC gel) was manufactured by DPT Laboratories, Ltd. (San Antonio, TX) under cGMP. Placebo vaginal film was manufactured by Par Pharma Inc. (Stratford, CT) under cGMP. Placebo vaginal ring was manufactured by Particle Science Inc. (Bethlehem, PA) under cGMP.

### Samples for determining feasibility

Samples for ex vivo testing were created by utilizing double headed rayon vaginal swabs (Starplex Scientific, Cleveland, TN) obtained from volunteers under protocol number #09-09-FB-0175, approved by the Eastern Virginia Medical School (EVMS) Institutional Review Board (IRB). Placebo vaginal gel, film, and insert were dissolved in water at different dilutions. The vaginal swabs were dipped briefly into the solution or diluted placebo product added directly to the swab before extraction. For determining whether semen interfered with placebo product detection, diluted semen was added to the vaginal swab with or without placebo product. Volunteers donated semen under the EVMS IRB approved protocol #13-02-FB-0031. For mimicking vaginally used IVRs, placebo IVRs were soaked at 37 °C for 1 and 5 days in either vaginal fluid simulant [24] for subsequent glycerin analysis or phosphate buffered saline (PBS) ± 10% fetal bovine serum (for analysis of penetrated bioanalytes) before extracting contents of the IVRs.

### CONRAD D15-135 study

The CONRAD D15-135 study was conducted at EVMS. The protocol was approved by the Chesapeake IRB (Pro00013292) and registered with ClinicalTrials.gov (#NCT02569697). We enrolled healthy, HIV-1 un-infected, women aged 18–50 years, not at risk for pregnancy. Study visits for the various product cohorts are outlined in Table 1.

**Table 1:** CONRAD 135 study design

Product	Visit 1	Visit 2	Visit 3	Visit 4	Visit 5
Intravaginal ring (IVR) (n = 20)	Screening	Insert IVR1 (24–36 h)	Remove IVR1 Vaginal swab Insert IVR2 (7–10 days)	Remove IVR2 Vaginal swab Insert IVR3 (28–32 days)	Remove IVR3 Vaginal swab
Gel, film, or insert (n = 10 each)	Screening	Baseline swab Apply product in clinic Swab 15 min post-product Swabs at home each day, Day 1–Day 7	Baseline swab Apply product in clinic Swabs 15 min post-product Unprotected intercourse 8–16 h post-product use Swabs at home each day, Day 1–Day 7	Final swab	N/A

In brief, women were screened at Visit 1 (V1) for exclusionary conditions. All participant assigned to use a placebo product were instructed to refrain from vaginal intercourse and vaginal product use at least 48 h prior to the first treatment visit (Visit 2, V2). All vaginal products were inserted in the clinic under direct observation. We obtained baseline double headed vaginal swabs prior to product use (negative control) and approximately 15 min after product use (positive control) in the clinic, at V2. Participants obtained vaginal swabs at home, nightly for 7 nights, starting on the night after initiating product use. All vaginal swabs for home use were affixed with pre-printed labels. Participants were instructed to abstain from vaginal intercourse until coming back to the clinic for Visit 3 (V3). At V3, nightly swabs from the previous week were collected, and point-of-care testing for prostate specific antigen (PSA) to exclude recent vaginal semen exposure (ABAcard, Abacus Diagnostics, West Hills, CA) was performed. Baseline vaginal swabs prior to product use and approximately 15 min after product use were collected in the clinic. Within approximately 12 h (± 4 h) after leaving the clinic, participants were asked to have unprotected, vaginal intercourse with their male partner and to repeat the nightly vaginal swab process, beginning after intercourse. They were instructed to not have additional acts of intercourse during that 7-day period. Participants stored re-sealed vaginal swabs in their refrigerator prior to returning them to the clinic at their next visit. At Visit 4, nightly swabs from the previous week were collected and one final swab was obtained in the clinic. Swabs were analyzed in the lab for placebo markers using excipient-based assays and ATR-FTIR.

For IVR participants, all IVRs were inserted and removed by the investigator at Visits 2–5. Although we instructed participants not to remove the IVR at home, we noted any expulsions/removals at each follow up visit. IVR1 was worn for approximately 24–36 h, IVR2 was worn for 7–10 days, and IVR3 was worn for 28–32 days. We asked participants to refrain from vaginal intercourse while using IVR1, but no restrictions were placed on intercourse during use of IVR2 or IVR3. Vaginal swabs were obtained prior to insertion of the new IVR. Participants could re-enroll in the trial to use a different placebo dosage form, as long as they met all inclusion criteria and did not develop any exclusion criteria. Participants could use up to all four dosage forms, but could not repeat study visits for the same dosage form.

### Swab and IVR extractions for the CONRAD D13-135 study

Placebo gel, film, and insert were extracted from one swab head of the double headed vaginal swabs collected from the participants by placing it in 500 µl deionized or distilled water for 5 min at room temperature. The swab was then placed in a spin basket (Fitzco, Inc., Spring Park, MN) and spun at 7500×*g* for 5 min to remove all remaining extract from the swab. This extract was then pooled with the initial extract for a final spin to remove insoluble particulates.

**Fig. 1:**
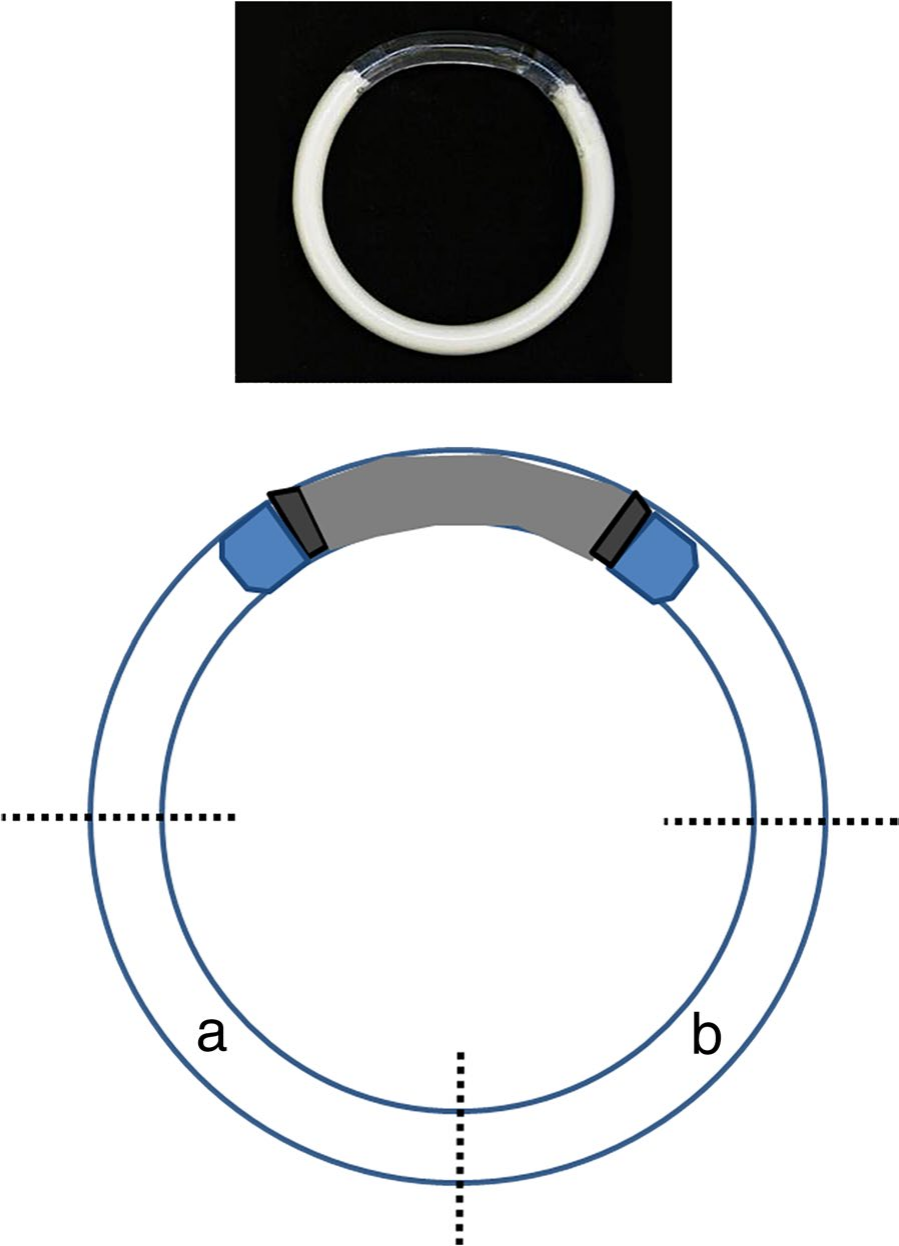
Schematic of IVR extractions. The IVR was cut horizontally across, and the resulting bottom half was cut into two portions. From one portion, (**a**) glycerin was extracted and from the other (**b**) bioanalytes were extracted. A photo of the IVR is shown on top

Ring contents were extracted from two equally cut segments of the hollow reservoir of the IVR for glycerin and bioanalyte analysis, respectively, as shown in Fig. 1. Another portion of the ring was sent to Northwestern University for additional analysis [25]. To extract glycerin, the IVR segment was cut into smaller pieces with the inner core material manually pushed out of the polyurethane tube (IVR segment’s reservoir sheath) into a 50 ml conical tube, containing 10 ml deionized water. The water extract was gently shaken for 3 h at room temperature. An aliquot of 1.5 ml was then removed and re-spun at 20,000×*g* for 5 min to further remove any insoluble particulates before analyzing for glycerin. To extract bioanalytes penetrated into the IVR, the segment was placed in a 15 ml tube containing 2 ml RIPA buffer (1% Igepal-630, 0.5% Deoxycholate, 0.1% SDS in PBS), a commonly used buffer for protein extraction. The inner core material was manually pushed out of the IVR into the solution, and the tube place on ice for 1 h with occasional shaking. The extracts were then spun at 2500 x g for 10 min at 4 °C. The supernatants were removed and spun one more time at maximum speed at 4 °C to pellet any further insoluble particulates before analysis of the bioanalytes.

### Excipient-based analyses of clinical vaginal swabs and IVR extracts

Glycerin present in water extracts of IVRs and vaginal swabs containing film was measured using an enzymatic, colorimetric assay according to manufacturer’s instructions (Sigma-Aldrich, St. Louis, MO). Sorbitol, an excipient component of the vaginal inserts, was measured in water extracts of vaginal swabs post vaginal insert use according to manufacturer’s instructions (BioAssay Systems, Hayward, CA).

Hydroxyethylcellulose (HEC), the major excipient in the placebo gel, was qualitatively measured utilizing CBM29-1-2, a polyhistidine (HIS)-tagged recombinant peptide probe obtained from PlantProbes (Leeds, UK). This probe-based detection was based on the original one described in a previous publication validating the probe’s affinity for HEC [26]. Briefly, water extracts of vaginal swabs containing placebo gel were spotted on a nitrocellulose membrane and allowed to dry. After blocking in 5% milk/PBS, the membrane was incubated with 1:80 of CBM29-1-2 in 5% milk/dH_2_O for 1 h at room temperature. After 3 washes in water, bound CBM29-1-2 was detected by using 1:1000 mouse anti-polyhistidine-peroxidase antibody (Sigma-Aldrich, St. Louis, MO) in 5% non-fat dry milk/PBS for 1 h. After 3 washes in water, detection was completed by incubating the membrane with an HRP substrate solution containing 10 ml of dH_2_O, 4 ml of methanol, 10 mg of 4-chloro-1-napthol (Sigma), and 0.024 ml 3% H_2_O_2_ to generate a bluish-purple color if HEC was present.

The concentration of bioanalytes that penetrated the IVRs were quantitated using the CBQCA Assay, a fluorescence-based total protein assay (Thermo-Fisher Scientific, Waltham, MA). The CBQCA reagent reacted with any biological material containing free amine groups in the IVR extracts to generate a fluorescent signal. Concentrations were calculated based on a standard curve using bovine serum albumin (BSA) according to manufacturer’s instructions.

### ATR-FTIR spectroscopy of vaginal swab-placebo extracts

**Fig. 2:**
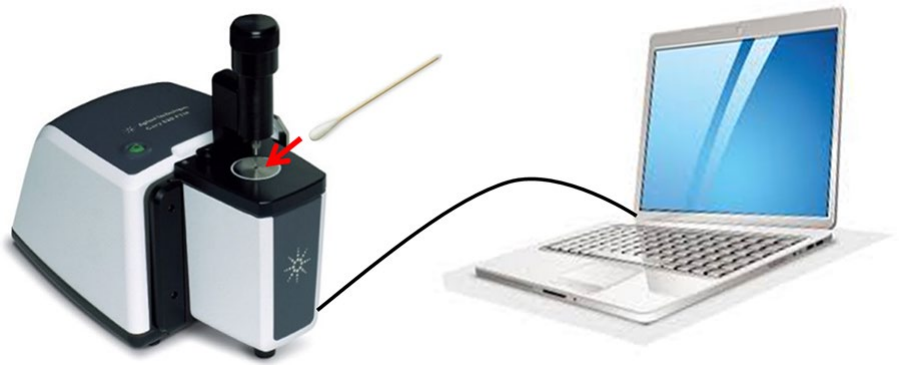
Measuring adherence by spectroscopy using the Agilent Cary 630 FTIR Spectrometer. A vaginal swab is placed directly on the “diamond” (red arrow) whereby a laser is projected onto the swab to generate a spectral scan. The spectral scan is analyzed through discriminant model algorithms on the laptop for a final “yes” or “no” output

Placebo product was detected in clinical samples through FTIR spectroscopy using methodology previously developed [21]. One swab head of the double headed vaginal swabs collected from participants was used to obtain FTIR spectra data by directly placing the swab on a portable Agilent Cary 630 FTIR Spectrometer equipped with a Diamond Head accessory. The operations of the FTIR equipment was controlled by Microlab PC software run from a dedicated computer laptop (Agilent Technologies Inc., Santa Clara, CA). The Microlab PC software was programmed to provide simple “yes” or “no” readouts based on discriminant algorithm predictions after direct and non-destructive analysis of a vaginal swab (Fig. 2).

### Vaginal insertion and semen exposure confirmation

One swab head of the double headed vaginal swabs collected from participants was used for DNA extraction to confirm vaginal insertion and semen exposure. DNA extracts were analyzed using a previously published multiplex PCR that amplified various vaginal bacteria to confirm vaginal insertion and two Y-chromosomal DNA markers, SRY and TSPY4, to confirm semen exposure [27].

### CONRAD 135 study data analysis

For demographic data (Tables 4 and 5), statistical analyses were performed with SAS version 9.3 (Cary, NC). Descriptive statistics included mean, median, standard deviation for continuous variables and frequencies and percentages for categorical variables. Continuous endpoints from the product cohorts were compared using an independent samples t- test for normally distributed data or Kruskal–Wallis test for non-normally distributed data. Normality of the data was assessed using the PROC UNIVARIATE command in SAS and examining the data distribution in the histogram, probe plot and the Shapiro Wilkes statistic. For categorical variables, Fisher’s exact test was used as indicated by expected cell size. Statistical significance was determined at the level of alpha = 0.05.

For vaginal gel, insert, and film swab extracts (Table 6), excipient and FTIR data were expressed as the percent of total swabs per time point from ten women that had positive detection of the placebo product. Each woman provided one swab per time point. For IVR adherence measures (Fig. 4), twenty women used one IVR during each timeframe. Differences among mean glycerin and bioanalyte values of twenty IVR extracts per time point were compared by Repeated Measures ANOVA, significance at p < 0.05.

## Results

### Feasibility of excipient-based measurements to determine topical vaginal placebo product use

The excipient-based approach for detecting placebo gel, film, and insert involved measuring that component contributing the largest percentage of the formulation and/or leveraging the availability of a sensitive assay specific for it. Glycerin and sorbitol were measured to detect vaginal film and insert, respectively. Glycerin was detected only in swabs containing vaginal film and not in vaginal swabs alone or with semen thereby confirming no potential for false positives by other substances in these biological fluids (Table 2).

**Table 2:** Measuring glycerin as a marker for the presence of vaginal film

Swab extract	Glycerin (mM)
Vaginal swab only	0.00
Vaginal swab + 1:5 film^a^	1.54
Vaginal swab + 1:25 film^a^	0.36
Vaginal swab + 1:100 film^a^	0.07
Vaginal swab + 1:5 semen^a^	0.00
Vaginal swab + 1:10 semen^a^	0.00

^a^Swabs were briefly dipped into various dilutions of film and semen

Unlike the glycerin data, endogenous levels of sorbitol were measured in vaginal swabs and semen. Therefore, sorbitol was tested in vaginal swabs from multiple women (Table 3). However, the presence of vaginal insert increased the amount of sorbitol quantitated in the vaginal swab extract greater than tenfold above background, baseline levels.

**Table 3:** Measuring sorbitol as a marker for presence of vaginal insert

Swab extract	Sorbitol (µM)
Swab only	1.9
Vaginal swab #1	41.8
Vaginal swab #2	35.3
Vaginal swab #1 + 50 µl insert^a^	660.9
Vaginal swab #2 + 50 µl insert^a^	294.7
Vaginal swab #3 + 100 µl insert^a^	1304.0
Semen—1:5	23.0
Semen—1:10	12.4
Semen—1:20	7.1
Semen 1:5 + 50 µl insert^a^	313.7

^a^One insert was dissolved in 1 ml of water before adding 50 or 100 µl to the vaginal swab

**Fig. 3:**
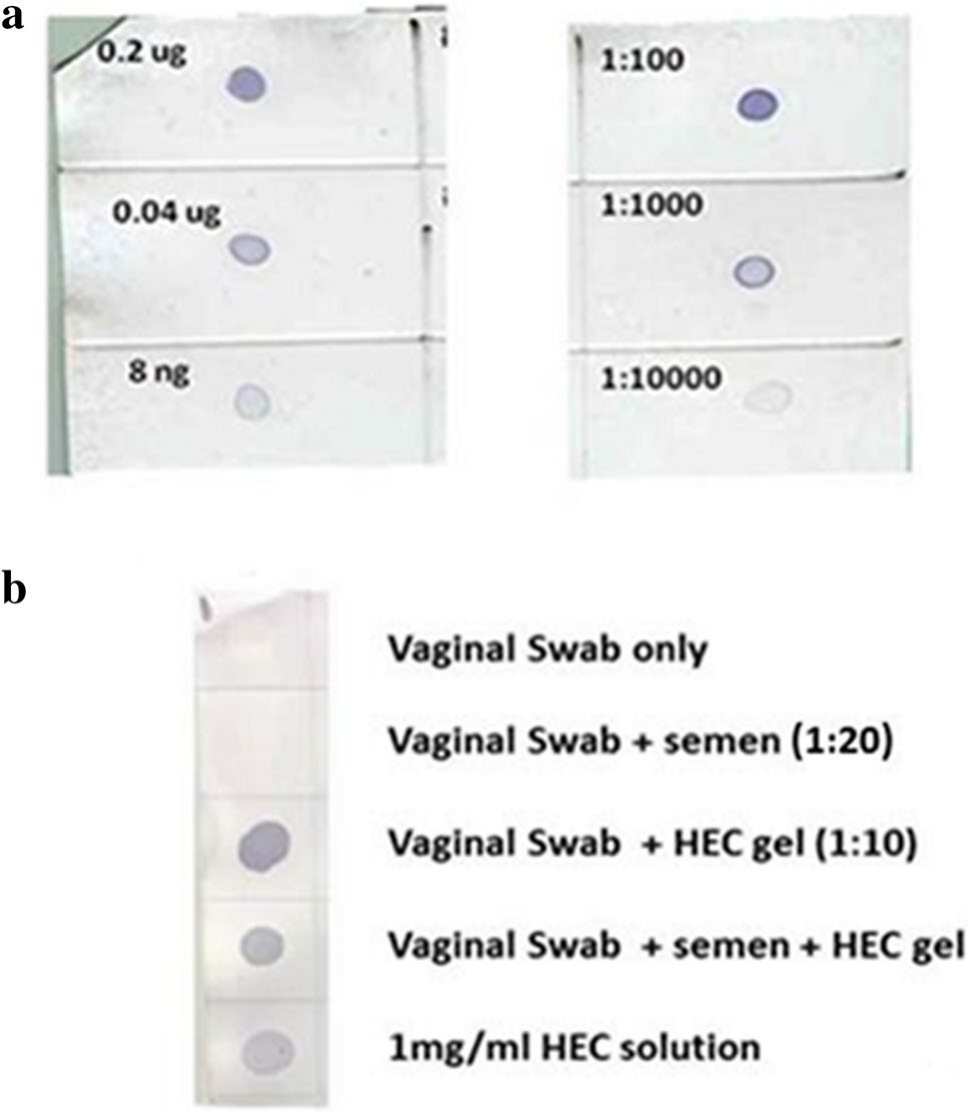
Validation of the CBM-29-1-2 probe in detecting hydroxyethylcellulose (HEC) in placebo vaginal gel. **a** Positive detection of various amounts of pure, dissolved HEC powder by the probe confirming the feasibility of the detection protocol (left). Positive detection of HEC in various dilutions of placebo vaginal gel (right). **b** Positive detection of HEC in vaginal swab extracts containing HEC gel ± semen. Extracts from vaginal swabs only or vaginal swabs containing semen are negative confirming specificity

Placebo HEC gel in vaginal swabs was detected by a probe-based, colorimetric approach. The probe detected pure HEC dissolved in water and placebo HEC gel at various dilutions (Fig. 3a) as well as HEC in extracts from vaginal swabs containing diluted placebo HEC gel. Semen did not interfere with HEC detection or cause false positive results when added to vaginal swabs (Fig. 3b).

Since IVR use involved a 30-day exposure, adherence markers for the IVR needed to correlate with elapsed time of exposure. Two approaches were investigated: an excipient-based marker that slowly elutes out of the IVR over time and a bioanalyte-based marker that slowly penetrates the IVR over time. We investigated glycerin, an excipient used in the hollow reservoir segment of the IVR intended to facilitate delivery of TFV as it elutes out of the IVR when water penetrates into it. We also investigated the possibility that small molecular weight biological material in vaginal fluid, as measured by the CBQCA assay, could slowly penetrate the IVR due to a concentration gradient generated between the inner compartment of the IVR and the vagina. While not a perfect in vivo simulation, soaking placebo IVRs in either vaginal fluid simulant solution or PBS containing 10% FBS as a source of small bioanalytes, demonstrated the proof of concept that glycerin levels inside the IVR decreased while the concentration of bioanalytes increased over the course of 5–7 days (Additional file 1: Table S1 and Additional file 2: Table S2).

### Clinical validation of the placebo adherence markers

To validate the feasibility results just described, the clinical study, CONRAD D15-135, was performed in which women were assigned to use the various placebo vaginal dosage forms. Demographics of these women are shown in Tables 4 and 5.

**Table 4:** Demographics of participants enrolled in the 135 study continuous variables

Variable	Film (n = 10)		Gel (n = 10)		Insert (n = 10)		IVR (n = 20)		P value^a^
	Mean	STD	Mean	STD	Mean	STD	Mean	STD	
Age (years)	36.5	4	37.5	4.3	34.8	6.8	36.3	6.8	0.87
Height (inches)	65.2	1.8	65.3	1.7	65.1	2	64.6	2.4	0.92
Weight (pounds)	179.4	34.7	181.3	35.9	176.6	37.4	179.9	46.7	0.99
Education (years)	15.1	1.8	14.4	1.9	14.8	1.9	14.2	2.6	0.67
Vaginal deliveries	1.4	1.4	1.3	1.3	1.6	1.4	1	1	0.74
Cesareans	0.9	1.2	1.2	1.1	0.6	0.9	0.8	0.9	0.63

^a^P values were generated using Kruskal–Wallis test

**Table 5:** Demographics of participants enrolled in the 135 study categorical variables

Variable	Film (n = 10)		Gel (n = 10)		Insert (n = 10)		IVR (n = 20)		P value^a^
	N	%	N	%	N	%	N	%	
Ethnicity									
Hispanic	1	10	0	0	2	20	3	15	0.77
Non-hispanic	9	90	10	100	8	80	17	85	
Race									
Black	3	30	5	50	3	30	7	35	0.89
White	6	60	5	50	5	50	10	50	
Other (mixed race, Asian)	1	10	0	0	2	20	3	15	
Use of exogenous hormones (OCPs, IUD, implant)									
Yes	7	70	6	60	8	80	5	15	0.01
No	3	30	4	40	2	20	15	85	

^a^P values were generated using Fisher’s exact test

This was not a randomized trial, so there were differences in contraceptive methods among participants electing to use the various placebo products. For vaginal placebo gel, film, and insert, one set of vaginal swabs were scanned for ATR-FTIR analysis. Spectral scans of those swabs were analyzed using discriminant models previously validated to specifically predict the presence or absence of each topical product. Excipients for each topical product were measured in water extracts from another set of vaginal swabs to determine whether placebo product could be detected in the samples. Table 6 is a summary of the percentage of total swabs from ten women (n = 9–10 swabs per time point, one swab per woman) containing placebo product at each time point as determined by excipient assay and ATR-FTIR analysis.

**Table 6:** Placebo product detection in vaginal swabs of the 135 Study

Time	HEC gel		Vaginal film		Vaginal insert	
	HEC	FTIR	Glycerin	FTIR	Sorbitol	FTIR
0	0	0	0	0	0	0
15 min	100	100	100	100	100	100
6–12 h	100	100	90	100	90	80
24 h	90	50	70	90	80	20
48 h	40	50	40	70	70	40
72 h	22	50	20	40	30	40
96 h	20	60	10	30	60	50
120 h	10	50	0	20	30	50
144 h	10	50	0	10	10	40
With semen exposure						
0	10	0	0	0	0	0
15 min	100	100	100	100	90	90
8–16 h^a^	100	70	70	90	90	100
24 h	50	70	40	100	60	50
48 h	20	30	30	80	40	30
72 h	30	40	20	50	30	30
96 h	20	10	0	30	10	20
120 h	20	10	0	40	0	30
144 h	0	20	0	40	0	22

Numbers represent the percent of total vaginal swabs containing detectable placebo product, **n = 9–10 swabs per time point**

^a^Swab obtained after vaginal intercourse

Both excipient assays and FTIR consistently detected product in 80–100% of vaginal swabs up to 12 h post-vaginal application of the placebo product without semen exposure. Semen exposure approximately 8–16 h after placebo use did not interfere with the detection of product in vaginal swabs (70–100%) during this same timeframe. The ability to detect product in the vaginal swabs in the presence or absence of semen began to decrease beginning at 24 h post-product use for both methodologies.

**Fig. 4:**
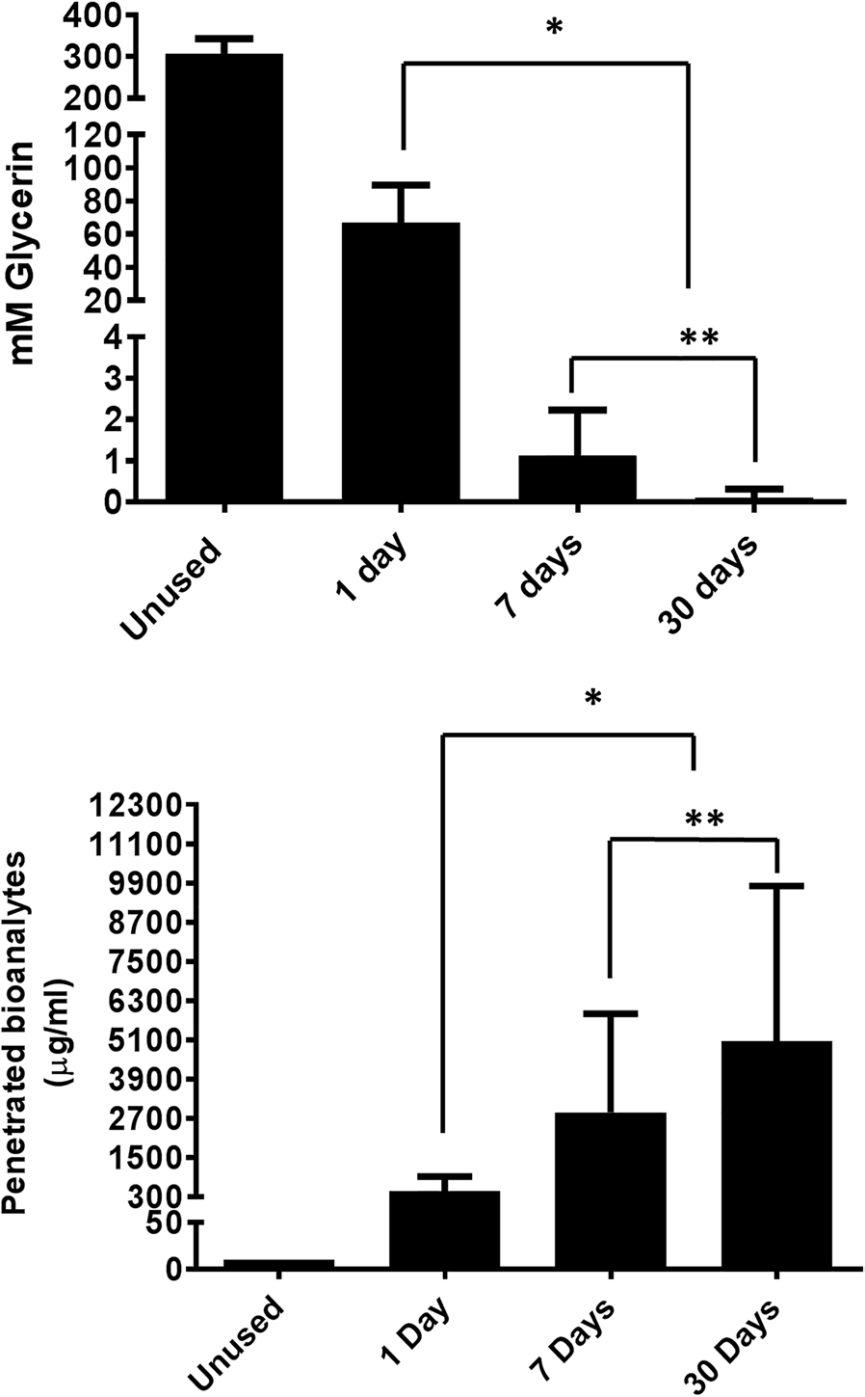
Using objective markers to assess IVR use in the CONRAD 135 Study. Twenty women used one IVR per time point. Glycerin (top) and penetrated bioanalytes (bottom) were measured in separate extracts, each representing one-quarter of an IVR. Glycerin and bioanalytes levels were significantly different among all time points. N = 20; *p < 0.0001, 1 day vs. 7 and 30 days, **p < 0.0001, 7 days vs. 30 days

Analysis of extracts for residual glycerin and penetrated amine-containing bioanalytes from collected IVRs confirmed that glycerin levels decreased while bioanalyte concentrations increased over the course of 30 days. Changes in the both glycerin and bioanalytes among IVRs used for approximately 1, 7, and 30 days were significantly different (Fig. 4). Even though the absolute values and kinetics of the clinical data differed with the in vitro feasibility data (Additional file 1: Table S1 and Additional file 2: Table S2), the expected directionality of the changes across time was confirmed.

## Discussion

In this report, we present markers of placebo use for four different vaginal dosage delivery systems using excipient-based and spectroscopy-based methodologies. Both approaches are easy to perform using only a vaginal swab and/or returned IVR, which allows flexibility for women to obtain a pain-free sample in their own home rather than in clinic. We also demonstrate clinical proof of concept for their feasibility and implementation in a clinical setting. Furthermore, these new methodologies have been used in an acceptability study of vaginal dosage forms as microbicides and multipurpose prevention technologies in Africa (Quatro Study, NCT# 02602366), allowing, for the first time, confirmation and validation of placebo product acceptability and use as well as participant’s product preferences.

Both methodologies detected placebo gel, insert, and film in swabs consistently up to 12 h post vaginal use followed by a time dependent decline in percent of vaginal swabs with detectable product. The ideal excipient is one that is physiologically inert, non-toxic, chemically stable, and non-reactive [28]. This relatively fast decay is expected since placebo products are mixtures of various excipients, which in many cases remain in the lumen of the compartment and are eliminated by natural discharge. In some samples, we could detect placebo product through excipient-based assays at much later time points not always decaying to zero, particularly sorbitol and HEC assays. Since sorbitol is found in vaginal fluid, the detection observed in swabs from the much later time points could reflect woman to woman variations in these endogenous levels that were higher than the range of levels observed in baseline swabs. The Quatro Study referenced above will provide more samples to better determine the cutoff sorbitol level for determining product use. HEC was detected by a recombinant probe based on a protein sequence common to a family of carbohydrates conferring its ability to detect other types [26]. Therefore, it is possible that other carbohydrates present in the vaginal fluid or vaginal products could be occasionally detected. This may also be the reason for the baseline detection of HEC in 10% of the total swabs. Overall, the CONRAD 135 study results presented in this report confirm that women can swab themselves within 12–24 h of placebo product administration with or without sex in their home for successful detection of use.

The results obtained using spectroscopy for detecting vaginal placebo products confirmed the feasibility of this approach as a potentially non-destructive method to measure adherence. Unlike the excipient-based assay approach, the vaginal swab does not need to be extracted; the spectral scan is obtained directly from the swab to provide quicker predictions of vaginal use. Similar to the excipient-based assay approach, ATR-FTIR spectroscopy positively detected placebo product within 12 h post vaginal application followed by a time dependent decay starting at 24 h. The decay does not decrease as consistently as that observed for the excipient assays. Unlike the excipient approach which targets only that specific component of the placebo formulation, ATR-FTIR spectroscopy analyzes all differences across a spectrum between vaginal swabs with or without placebo. Therefore, non-specific changes in the vaginal fluid could contribute to possible false negatives or positives. However, detecting all differences between the two samples rather than a single excipient is advantageous if vaginal products such as lubricants containing the same excipient are present. In this case, the excipient assay could generate a false positive result while the spectroscopy approach predicts correctly because the spectral scan of the lubricant is different from the one representing the placebo product. The spectrometer has a small window of detection generating a scan from a portion of the swab thereby requiring multiple scans to make a prediction. This could be a source of variability in the results of the ATR-FTIR analysis. We are actively working to improve the discriminant prediction models to decrease variability. While different types of infrared spectroscopy are commonly used in identifying non-biological material, its use in differentiating body fluids, a more complex matrix with donor to donor variability, is still under much investigation [29, 30]. In summary, with improvement to both approaches, the two methods can eventually be complementary. The quick and easy approach by FTIR could be the first round of measuring placebo use, followed by confirmation, if necessary, by the specific excipient assay.

The IVR can theoretically deliver topical products with improved adherence since it only requires a one-time insertion for the appropriate therapeutic window, thereby removing the inconvenience of event-driven approaches such as gels, inserts, and films. However, we have learned through previous studies that the IVR is subject to imperfect use that cannot be directly observed [31]. Therefore, an objective marker that changes over time would help determine if the woman removed the IVR during a period of use. We have presented two measures, glycerin elution and bioanalyte penetration, that significantly changed during the course of 30-day use. Our data demonstrates that some bioanalytes in vaginal fluid are small enough to slowly enter the IVR with statistically significant increases over time, albeit with high variability among women. While glycerin elution is less variable and very specific to the placebo IVR used in the study, the concept of vaginal bioanalyte penetration could theoretically apply to other types of IVRs. Further investigation using a proteomic approach may help to identify a specific analyte in the extract that can serve as a more consistent adherence marker. Psychometric measures are also being developed for assessing non-compliant intravaginal IVR use as well as predicting which women in a HIV prevention trial may need additional support and intervention for maintaining appropriate compliance during the study [31]. Placebo IVR studies with objective markers could help to further this type of sociobehavioral investigation.

The adherence necessary to achieve high efficacy of prevention involves appropriate drug levels, protocol compliance, and persistence (continued use during the total time of the high risk activity) [1]. Attaining good adherence for PrEP can be difficult because behaviors and lifestyles can change when high risk events arise. Ideally, protocol compliance and persistence must be adaptable to these changes. The placebo arm of an HIV PrEP study may not only serve as a negative control for determining drug efficacy but may also help to determine whether differential side effects are reflections of use differences within the active arm as well as identify possible differences in risk exposure associated with levels of adherence. To draw conclusions such as these, markers of adherence must be present and measured consistently within both placebo and active arms of a clinical trial. While the present study focused on clinical proof-of-concept testing of placebo vaginal product adherence measures only, additional studies are necessary to determine successful detection of the markers in the presence of active drug with or without semen. However, our primary and specific goal was to develop placebo adherence markers and confirm feasibility of successful detection in preparation for their use in the Quatro Study, a large product acceptability study in Africa for these specific vaginal placebo products.

An objective adherence marker that is consistently and equally measured in both placebo and treatment groups, would also support statistical inferences on drug efficacy in the context of different levels of adherence, thus leading to improved interpretation of trial results, especially within the context of subgroup analysis and alternative trial designs. Even though the utility and ethical issues of placebo-controlled effectiveness trials have been debated [32], placebos help to assess adherence and acceptability of products before launching into resource-intense effectiveness trials. For example, placebo practice trials are considered an approach to help predict adherence readiness for antiretroviral therapy by drug users [33]. There are also observations that good adherence not only to drug therapy but also to placebo is associated with lower mortality risk [34]. This result implies that adherence to drug therapy can be associated with healthy behaviors, termed the “healthy adherer effect”. In summary, drug-independent markers that objectively measure adherence in the placebo and active arms of an ARV-based HIV prevention study with the same sensitivity and specificity could lead to better assessment of product use, behaviors, and product acceptability. This more objective adherence data would further inform the development of alternative trial designs based on adherent population enrichment techniques.

## Conclusions

We have provided clinical proof of concept for the development of objective adherence markers of placebo-based vaginal gel, film and insert. We have also discovered two potential time-dependent markers of IVR use. In conclusion, our data confirms development of drug-independent, real-time, objective markers of adherence that can be used in a clinic at lower costs. These objective placebo markers of adherence have been used successfully in a large acceptability study of placebo vaginal delivery forms for preventing HIV in women in Africa (Quatro Study, NCT# 02602366).

## Additional files

**Additional file 1: Table S1.** In vitro residual glycerin levels in placebo IVRs.

**Additional file 2: Table S2.** In vitro quantitation of bioanalytes penetrated into IVRs.

## Abbreviations

ARV: anti-retroviral
PrEP: pre-exposure prophylaxis
LC–MS/MS: liquid chromatography tandem mass spectrometry
HEC: hydroxyethylcellulose
TFV: tenofovir
IVR: intravaginal ring
ATR-FTIR: attenuated total reflectance Fourier transform infrared spectroscopy
PSA: prostate specific antigen
HIS: histidine
SRY: sex determining region of the Y chromosome
TSPY4: testis-specific protein
