# Supplementary figures and images for: Development and clinical assessment of new objective adherence markers for four microbicide delivery systems used in HIV prevention studies
Source: Clin Transl Med. 2018 Nov 7;7:37. doi: 10.1186/s40169-018-0213-6 (PMC6219998; doi:10.1186/s40169-018-0213-6)

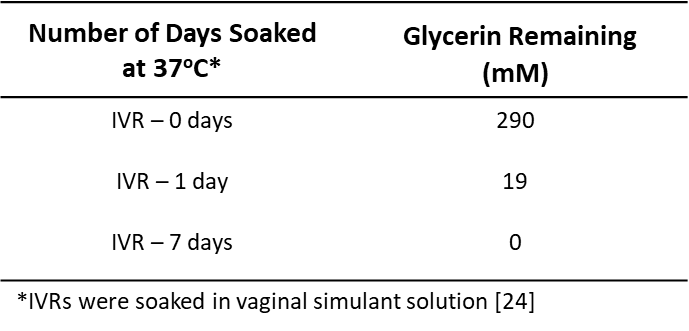


**Table S1. In Vitro Residual Glycerin Levels in Placebo IVRs**

Supplement: Supplementary file 1 — Additional file 1: Table S1. In vitro residual glycerin levels in placebo IVRs. [file 40169_2018_213_MOESM1_ESM.docx]

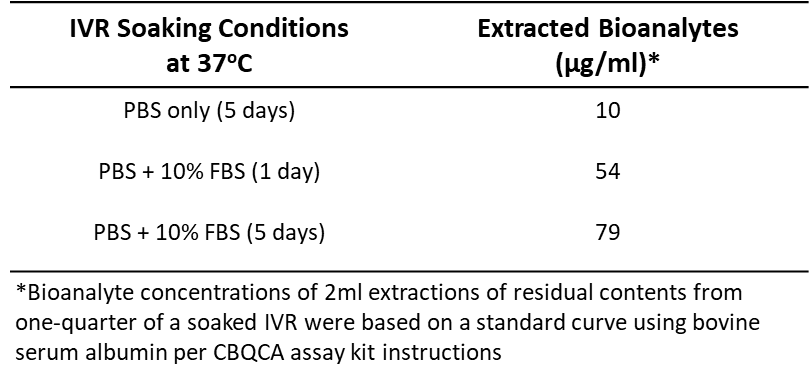


**Table S2. In Vitro Quantitation of Bioanalytes Penetrated into IVRs**

Supplement: Supplementary file 2 — Additional file 2: Table S2. In vitro quantitation of bioanalytes penetrated into IVRs. [file 40169_2018_213_MOESM2_ESM.docx]
